# *In vivo* effects of rosiglitazone in a human neuroblastoma xenograft

**DOI:** 10.1038/sj.bjc.6605506

**Published:** 2010-01-12

**Authors:** I Cellai, G Petrangolini, M Tortoreto, G Pratesi, P Luciani, C Deledda, S Benvenuti, C Ricordati, S Gelmini, E Ceni, A Galli, M Balzi, P Faraoni, M Serio, A Peri

**Affiliations:** 1 Endocrine Unit, Department of Clinical Physiopathology, Center for Research, Transfer and High Education on Chronic, Inflammatory, Degenerative and Neoplastic Disorders for the Development of Novel Therapies (DENOThe), University of Florence, 50139 Florence, Italy; 2 Department of Experimental Oncology and Laboratories, Fondazione IRCCS Istituto Nazionale Tumori, 20133 Milan, Italy; 3 Department of Veterinary Pathology, Hygiene and Public Health-Section of Veterinary and Avian Pathology-School of Veterinary Medicine, University of Milan, 20133 Milan, Italy; 4 Clinical Biochemistry Unit, Department of Clinical Physiopathology, Center for Research, Transfer and High Education on Chronic, Inflammatory, Degenerative and Neoplastic Disorders for the Development of Novel Therapies (DENOThe), University of Florence, 50139 Florence, Italy; 5 Gastroenterology Unit, Department of Clinical Physiopathology, Center for Research, Transfer and High Education on Chronic, Inflammatory, Degenerative and Neoplastic Disorders for the Development of Novel Therapies (DENOThe), University of Florence, 50139 Florence, Italy; 6 Radiation Biology Unit, Department of Clinical Physiopathology, Center for Research, Transfer and High Education on Chronic, Inflammatory, Degenerative and Neoplastic Disorders for the Development of Novel Therapies (DENOThe), University of Florence, 50139 Florence, Italy

**Keywords:** PPAR*γ*, RGZ, NB, xenograft

## Abstract

**Background:**

Neuroblastoma (NB) is the most common extra-cranial solid tumour in infants. Unfortunately, most children present with advanced disease and have a poor prognosis. There is *in vitro* evidence that the peroxisome proliferator-activated receptor *γ* (PPAR*γ*) might be a target for pharmacological intervention in NB. We have previously demonstrated that the PPAR*γ* agonist rosiglitazone (RGZ) exerts strong anti-tumoural effects in the human NB cell line, SK-N-AS. The aim of this study was to evaluate whether RGZ maintains its anti-tumoural effects against SK-N-AS NB cells *in vivo*.

**Methods and results:**

For this purpose, tumour cells were subcutaneously implanted in nude mice, and RGZ (150 mg kg^−1^) was administered by gavage daily for 4 weeks. At the end of treatment, a significant tumour weight inhibition (70%) was observed in RGZ-treated mice compared with control mice. The inhibition of tumour growth was supported by a strong anti-angiogenic activity, as assessed by CD-31 immunostaining in tumour samples. The number of apoptotic cells, as determined by cleaved caspase-3 immunostaining, seemed lower in RGZ-treated animals at the end of the treatment period than in control mice, likely because of the large tumour size observed in the latter group.

**Conclusions:**

To our knowledge, this is the first demonstration that RGZ effectively inhibits tumour growth in a human NB xenograft and our results suggest that PPAR*γ* agonists may have a role in anti-tumoural strategies against NB.

Neuroblastoma (NB) is a neoplastic disease of the sympathetic nervous system, derived from neuronal crest cells, and represents the second most common extra-cranial tumour in childhood and accounts for more than 7% of malignancies in patients younger than 15 years (National Cancer Institute, Surveillance, Epidemiology and End Results Database, 2005). Neuroblastoma is characterised by a heterogeneous clinical presentation and course (Maris *et al*, 2007), but the prognosis is often severe (around 15% of all paediatric oncology deaths) (Joshi and Tsongalis, 1997). In fact, most children with NB present with advanced disease and metastases at diagnosis and, despite intensive therapy, at least 60% of patients with high-risk features have a poor prognosis and high relapse chance (Matthay *et al*, 1999; De Bernardi *et al*, 2003). Thus, the identification of new and more effective therapies would be of pivotal importance to improve the outcome of affected children.

Retinoids have been shown for instance to interfere with cell growth and to induce apoptosis in NB cells (Melino *et al*, 1997; Voigt and Zintl, 2003), and in clinical trials they effectively increase event-free survival in high-risk patients, with limited toxic effects (Garaventa *et al*, 2003; Reynolds *et al*, 2003). Thiazolidinediones (TZDs), a class of molecules that activate the nuclear receptor peroxisome proliferator-activated receptor *γ* (PPAR*γ*) (Desvergne and Whali, 1999), promote association with the 9-*cis* retinoic X receptor to form functional heterodimers that recognise specific DNA response elements within target genes (Jude-Aubry *et al*, 1997; Reginato *et al*, 1998). The PPAR*γ*, initially described in adipose tissue (Tontonoz *et al*, 1993), is classically related to adipocyte turnover (Spiegelman, 1998), glucose and lipid metabolism regulation (Saltiel and Olefsky, 1996; Koeffler, 2003). In addition, a role of PPAR*γ* against inflammation, atherosclerosis and tumoural growth has been described (Jiang *et al*, 1998; Ricote *et al*, 1998; Kersten *et al*, 2000). In particular, PPAR*γ* signalling activation inhibits cell proliferation and/or induces apoptosis (Grommes *et al*, 2004). As a consequence, PPAR*γ* has been regarded as a possible target for anti-cancer therapy and TZDs have been used in clinical trials for the treatment of tumours involving different organs and tissues, such as prostate (Hisatake *et al*, 2000; Mueller *et al*, 2000), colon (Kulke *et al*, 2002), breast (Burstein *et al*, 2003), lung and adipose tissue (Demetri *et al*, 1999; Tsubouchi *et al*, 2000; Debrock *et al*, 2003).

Abundant PPAR*γ* expression has been detected in different tumours affecting the nervous system, such as astrocytomas (Chattopadhyay *et al*, 2000), gliobastomas (Nwankwo and Robbins, 2001; Morosetti *et al*, 2004) and NB (Han *et al*, 2001). In the recent past, it has been observed that in different NB cell lines expressing PPAR*γ*, endogenous or synthetic ligands induce anti-neoplastic effects such as cell differentiation, apoptosis, growth arrest, reduced viability and inhibition of invasiveness (Emmans *et al*, 2004; Servidei *et al*, 2004; Valentiner *et al*, 2005; Cellai *et al*, 2006; Peri *et al*, 2008).

In particular, we have previously shown that PPAR*γ* agonist rosiglitazone (RGZ) exerts strong anti-neoplastic effects (i.e., inhibition of cell proliferation and cell viability, decrease of matrix metalloproteinase-9 (MMP-9) expression, inhibition of cell adhesion and invasiveness) in SK-N-AS but not in SH-SY5Y human NB cells (Cellai *et al*, 2006). We also demonstrated that the different efficacy of RGZ was related to the presence of a significantly higher transcriptional activity of PPAR*γ* in SK-N-AS compared with SH-SY5Y, which was most likely because of a markedly higher amount of phosphorylated, that is, inactive, PPAR*γ* in the latter cell line. The aim of this study was to evaluate whether RGZ maintains its anti-tumoural effects against SK-N-AS NB cells *in vivo*. For this purpose, tumour cells were subcutaneously implanted in nude mice, and the effects of RGZ treatment against tumour growth, angiogenic activity and apoptosis induction were investigated.

## Materials and methods

### *In vivo* studies

Rosiglitazone maleate was provided by Vinci Biochem, Vinci, Italy. For *in vivo* studies, RGZ was dissolved in glycine/HCl buffer at pH 2.3, at a concentration of 7.5 mg ml^−1^. Anti-tumour activity experiments were carried out using 8–11-week-old female athymic Swiss nude mice (Charles River, Calco, Italy). Mice were maintained in laminar flow rooms with constant temperature and humidity. Experimental protocols were approved by the Ethics Committee for Animal Experimentation of the Fondazione IRCCS Istituto Nazionale Tumori (Milan, Italy), according to the United Kingdom Coordinating Committee on Cancer Research Guidelines (Workman *et al*, 1988).

Human NB SK-N-AS was maintained *in vivo* by serial subcutaneous passages of tumour fragments (about 2 × 2 × 6 mm) in healthy mice, as previously described (Pratesi *et al*, 1985).

For anti-tumour activity studies, each experimental group (i.e., solvent treated or RGZ treated) included 16 mice bearing a subcutaneous tumour in the right flank. Tumour fragments were implanted on day 1 and tumour growth was followed by bi-weekly measurements of tumour diameters using a Vernier caliper. Tumour weight (TW) was calculated according to the formula TW (mg)=tumour volume (mm^3^)=d^2^ × D/2, where d and D are the shortest and the longest diameters, respectively. Drug treatment started at day 1, shortly after tumour implant. RZG was delivered p.o. by gavage in a volume of 20 ml kg^−1^ of body weight, at a dose of 150 mg kg^−1^ according to a daily schedule (weekend excluded) for 4 weeks, for a total of 20 treatments. Control mice were treated in parallel with glycine/HCl buffer. Rosiglitazone treatment was stopped after 4 weeks; at this time, control tumours had reached about 2 g of weight, which is considered the maximum tolerated for ethical reasons. Drug efficacy was assessed as tumour weight inhibition % (TWI%)=100–(mean TW RGZ-treated mice/mean TW control mice × 100), evaluated during and after drug treatment. Drug tolerability was assessed in tumour-bearing mice in terms of (a) lethal toxicity, that is, any death in RGZ-treated mice occurring before any death in control mice; (b) body weight loss percentage=100–(body weight on day x/body weight on day 1 × 100), where x represents a day after or during the treatment period. Experimental groups were killed by cervical dislocation the day after the last treatment (day 26). Half of RGZ-treated and control mice were killed after 2 weeks for immunohistochemical or gene expression analyses (as detailed below).

### Immunohistochemical studies

After killing the mice, tumours were excised. Half of each tumour was fixed in 10% buffered formalin and the other half in zinc fixative. Samples were fixed for 24 h, embedded in paraffin, sectioned at 4 *μ*m and stained with standard haematoxylin and eosin or processed for immunohistochemical analysis. For caspase-3 and CD31 immunohistochemistry, sections were deparaffinised, rehydrated and treated with 3% hydrogen peroxide in distilled water for 20 min. The sections were labelled by the avidin–biotin–peroxidase procedure (Hsu *et al*, 1981) with a commercial immunoperoxidase kit (Vectastain Standard Elite; Vector Laboratories, Burlingame, CA, USA). Primary antibodies were applied at 4°C overnight. The reaction was revealed by incubating the sections with 3,3 diaminobenzidine (Vector Laboratories) for 1 min; the sections were counterstained for 1 min with Mayer's haematoxylin. Rabbit polyclonal anti-cleaved caspase-3 (Asp175) antibody (Cell Signaling Technology, #9661, Danvers, MA, USA) was applied on formalin-fixed sections at a working dilution of 1 : 2500. For cleaved caspase-3 evaluation, four hot spots of each sample were randomly selected at × 100 magnification and the number of cleaved caspase-3-positive cells was assessed in a × 400 microscopic field within each hot spot. All the hot spots selected were distributed along the interface between viable tumoural structures and necrotic areas.

To establish microvessel density, zinc-fixed sections from each tumour were immunostained with the rat monoclonal anti-mouse CD-31 antibody, MEC 13.3 (PharMingen, San Diego, CA, USA), a specific marker for endothelial cells (Vecchi *et al*, 1994), applied at a working dilution of 1 : 50. For each sample, CD-31-positive microvessels were counted in four × 100 microscopic fields randomly selected within viable tumoural areas.

For the immunohistochemical evaluation of Ki-67 expression, tumour samples fixed in buffered formalin were cut into 5-*μ*m-thick sections. Next, the sections were deparaffinised in xylol for 30 min, rehydrated in ethanol and washed in buffered saline solution (phosphate-buffered saline) at pH 7.4. For antigen recovery, slides were placed in racks containing 10 mM citric acid (pH 6.0) and heated in a microwave oven for 25 min at maximum power. The slides were then allowed to cool for 15 min in water and washed in phosphate-buffered saline. Subsequently, the sections were treated for 5 min with peroxidase block reagent (Dako, Glostrup, Denmark) to block endogenous peroxidases. Incubation with primary mouse anti-Ki-67 monoclonal antibody (clone MIB1, Dako) for 1 h (dilution 1 : 50) preceded the application of the avidin–biotin system ENVISION+ system-HRP (Dako) and visualisation was performed using 3,3 diaminobenzidine (Dako) as chromogen. Finally, the slides were washed with distilled water, counterstained with haematoxylin, dehydrated with ethanol and mounted with coverslips. Cells that expressed the Ki-67 protein were identified by the dark brown colouring of the nucleus. For each sample, Ki-67-positive cells were counted in eight × 200 microscopic fields randomly selected within viable tumoural areas.

The image analysis of each immunostaining was performed using Image-Pro Plus Software (Bethesda, MD, USA).

### Quantitative real-time reverse transcriptase PCR

Total RNA to be subjected to reverse transcription was extracted from tumour specimens. Total RNA isolation and cDNA synthesis were performed as previously reported (Luciani *et al*, 2004).

Primers and probe for PPAR*γ* was Assay-On-Demand gene expression products (Hs00234592_m1; Applied Biosystems, Foster City, CA, USA). PCR mixture (25 *μ*l final volume) consisted of 1 × final concentration of Assay-On-Demand mix, 1 × final concentration of Universal PCR Master Mix (Applied Biosystems) and 20 ng cDNA.

Measurement of MMP-9 and tissue inhibitor of matrix metalloproteinase-1 (TIMP-1) mRNA was performed using a multiplex quantitative real-time reverse transcriptase PCR method, using primers and probes as previously described (Cioppi *et al*, 2004). Each measurement was carried out in triplicate. According to the manufacturer's instructions (Applied Biosystems), PPAR*γ* mRNA quantitation was based on the comparative cycle threshold (*C*_t_) method using ribosomal 18S RNA expression for normalisation. The results were expressed as (2^−ΔΔCt^) × 10^3^.

### Western blot analysis of PPAR*γ* expression

The PPAR*γ* expression was determined as previously described (Cellai *et al*, 2006). Briefly, tissue samples were dissolved in lysis buffer. A volume of 30 *μ*g of protein was diluted in 2 × Laemmli's reducing sample buffer, incubated at 95°C for 5 min and loaded onto a 10% polyacrylamide–bisacrylamide gel. Thereafter, proteins were electroblotted into nitrocellulose (Sigma Co., St. Louis, MO, USA). Equivalent protein loading was verified by staining parallel gels with Coomassie R and by using tubulin as a reference protein. After blocking (5% skimmed milk for 2 h), nitrocellulose membranes were washed and then immunostained with a rabbit anti-human PPAR*γ* antibody (1 : 1000) (Santa Cruz Biotechnology, Santa Cruz, CA, USA) or with an anti-phospho-PPAR*γ* (Ser82) clone AW 504 (1 : 1000) (Upstate, Lake Placid, NY, USA), followed by a secondary anti-rabbit IgG antibody (1 : 2000) (New England Biolabs, Beverly, MA, USA). The antibody-reacted proteins were revealed by LumiGLO chemiluminescent reagent and peroxide (New England Biolabs).

### PPAR*γ* phosphorylation assay

The assay was performed as previously described (Cellai *et al*, 2006). Briefly, tissue samples were boiled and dissolved in 10% glycerol, 2% SDS, and 50 mM Tris–HCl (pH 7.5) for 10 min. The lysate was immunoprecipitated with a rabbit anti-human PPAR*γ* antibody (1 : 100) (Santa Cruz Biotechnology) at 4 °C for 3 h. Immunocomplexes were recovered by incubation with Protein-A Sepharose (Sigma Co.) for an additional 16 h at 4 °C. The immunocomplexes were then dissociated by boiling for 5 min in Laemmli's buffer, the beads were collected by centrifugation and SDS–polyacrylamide gel electrophoresis was performed with the supernatant. The proteins were electroblotted into nitrocellulose transfer membrane Protean (Whatman, VWR International, Milan, Italy) and were detected by incubating the filter with the anti-PPAR-*γ* antibody or with an anti-phosphoserine mouse monoclonal antibody (1 : 400) (clone PSR-45, Sigma Co.) followed by a secondary anti-rabbit IgG (1 : 2000) or anti-mouse IgG (1 : 2000) antibody, respectively (Amersham Biosciences, Little Chalfont, Buckinghamshire, UK). To verify equivalent protein loading, tubulin was chosen as the reference protein. Detection of the protein bands was performed using the Amersham ECL plus Kit (Amersham Biosciences).

### Statistical analysis

Data from anti-tumour activity studies were analysed using two-way analysis of variance, followed by *post hoc* Bonferroni test. Differences in immunohistochemistry studies were compared by Student's *t-*test (two tailed). The Spearman's rank correlation analysis was used to determine the relationship between TW and cleaved caspase-3 immunopositive cells. *P*-values <0.05 were considered as statistically significant. Analyses were performed with Graph Pad Prism, version 4.0 (Graph Pad Software Inc., San Diego, CA, USA).

## Results

### Effect of RGZ on tumour growth

**Figure 1:**
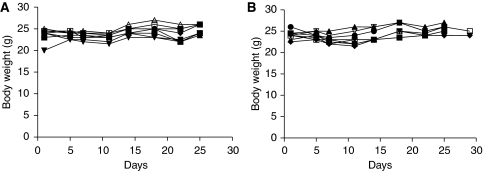
Mean body weight of mice during the treatment period. (**A**) Solvent-treated mice; (**B**) rosiglitazone (RGZ)-treated mice.

**Figure 2:**
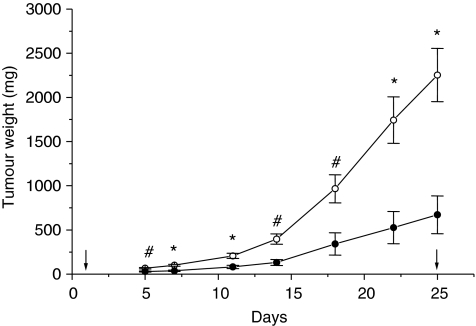
Growth curves of the SK-N-AS neuroblastoma (NB) xenograft. Tumour weight over time in solvent-treated (open circles) and 150 mg kg^−1^ rosiglitazone (RGZ)-treated (filled circles) mice. Treatments were delivered daily, by gavage, for 4 weeks (except weekend). Arrows indicate the first and last day of treatment. Each point represents the average±s.e. of at least eight tumours. ^*^P<0.005 and ^#^*P*<0.01 (solvent-treated *vs* RGZ-treated mice) by analysis of variance, followed by Bonferroni test.

For anti-tumour activity studies, RGZ was delivered p.o. by gavage at a dose of 150 mg kg^−1^ daily (weekend excluded) for 4 weeks against the SK-N-AS human NB tumour xenograft. The drug was well tolerated without lethal toxicity and body weight loss during treatment (Figure 1). Plasma glucose levels did not significantly differ at baseline or at the end of treatment (4 weeks) (88±7.73 *vs* 104.75±2.93 mg per 100 ml, mean±s.e., respectively, *P*>0.05). At the end of RGZ treatment, a marked TW inhibition (70%) was observed, compared with control mice (TW 670±214 *vs* 2250±300 mg, mean±s.e., respectively, *P*<0.005, Figure 2 and Table 1). Moreover, in the RGZ-treated group, three out of eight tumours were very small (<50 mg). These mice were not killed immediately after the end of RGZ administration and were subjected to an additional follow-up. One of the three tumours did not re-grow for up to 100 days (when the mouse was killed), and for the other two tumours, re-growth was observed 2 weeks after the end of treatment.

### Effect of RGZ on apoptosis and proliferation

To investigate the cellular mechanisms underlying RGZ activity, growing tumours from control and RGZ-treated mice were removed and fixed for immunohistochemistry analysis after 2 (half of the tumours/group) and 4 weeks of treatment. Histologically, by standard haematoxylin and eosin staining, control and RGZ-treated tumours seemed to be composed of sheets of dense to sparsely packed cells divided by septa of fibrovascular stroma. Peritumoural lymphoplasmacytic infiltrates were present (not shown).

**Figure 3:**
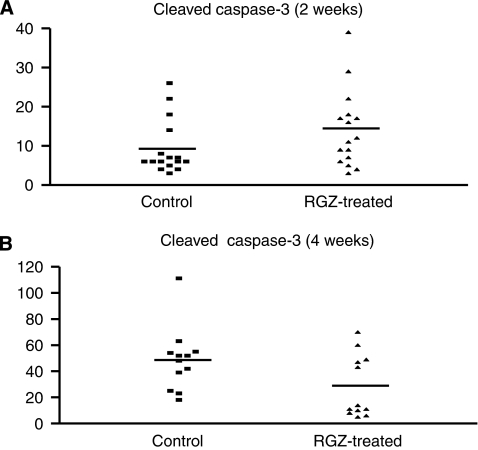
Immunohistochemistry (IHC) for cleaved caspase-3 assessment in tumour tissues from control (i.e., solvent treated) and rosiglitazone (RGZ)-treated (**A**, 2 weeks; **B**, 4 weeks) mice. *Y* axis: number of cleaved caspase-3-positive cells per field.

**Figure 4:**
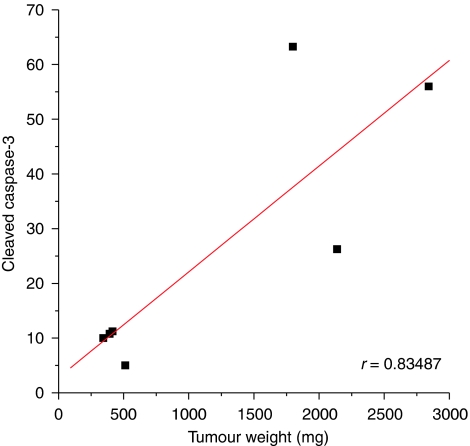
Relationship between tumour weight and cleaved caspase-3 immunopositive cells in SK-N-AS neuroblastoma xenografts. Black squares indicate solvent-treated tumours. *R*=0.835, *P*<0.05, by Spearman's rank correlation test.

With regard to apoptosis, at short-time observation (2 weeks), that is, in the presence of small-sized tumours, a low number of apoptotic cells was present, but in mice treated with RGZ, the number of cleaved caspase-3 immunopositive cells was higher than in controls, although the difference was not statistically significant (*P*=0.12) (Table 1 and Figure 3A). At such a time, necrosis was virtually absent in RGZ-treated tumours and rare and very limited necrotic areas were present in control tumours. A different pattern was observed in samples from mice killed after 4 weeks of treatment, in which, in large-sized control tumours (mean TW>2 g), large necrotic areas and a high number of apoptotic cells were observed. At such a time, in RGZ-treated tumours, the number of caspase-3 immunopositive cells was significantly lower (*P*<0.05) and necrotic areas were less extended than in solvent-treated tumours (Table 1 and Figure 3B). Figure 4 shows that, in control mice, a linear relation (*R*=0.835, *P*<0.05, by Spearman's rank correlation test) between positive staining for cleaved caspase-3 and tumour size was found. These results indicate that at the 2-week observation time, RGZ treatment increases the apoptotic index, whereas at 4 weeks, necrosis and apoptotic index are likely to be related to tumour size. Nevertheless, if we compare the rate of apoptosis in tumour samples of the same size from solvent-treated or RGZ-treated mice, we can observe that when in both groups the TW reached 670 g (i.e., the mean weight of tumours in RGZ-treated mice at the end of treatment, approximately corresponding to the weight of tumours in control mice after 2 weeks), the mean percentage of apoptotic cells was 10% in control mice, whereas it reached 30% in RGZ-treated mice (Figures 2 and 3).

**Figure 5:**
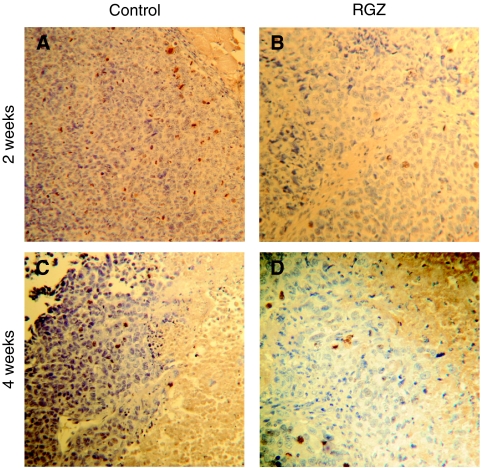
Immunohistochemical evaluation of Ki-67 in tumours from solvent-treated and rosiglitazone (RGZ)-treated mice (2, **A**–**B**, and 4, **C**–**D**, weeks) ( × 200 magnification). Necrotic areas are present on the right-end side of **C** and, to a much lesser extent, in **D**.

The proliferation rate of tumoural cells was also assessed, by determining the immunostaining for the Ki-67 index. After 2 weeks, the mean percentage of Ki-67-positive cells was moderately lower in RGZ-treated mice than in control mice (1.92±0.07 *vs* 2.17±0.29, mean±s.e., *P*=0.44). Such a difference, yet non-statistically significant, was maintained also at the end of the treatment period (4 weeks) (1.6±0.27 *vs* 2.25±0.43, mean±s.e., *P*=0.26). A representative example is shown in Figure 5. It has to be said that the same above-mentioned observations regarding the extension of necrotic areas at the end of the treatment period, particularly in tumours of control mice, may also very likely be applicable in explaining the absence of a more pronounced difference of the percentage of Ki-67-positive cells between the two groups of animals.

### Effect of RGZ on angiogenesis

**Figure 6:**
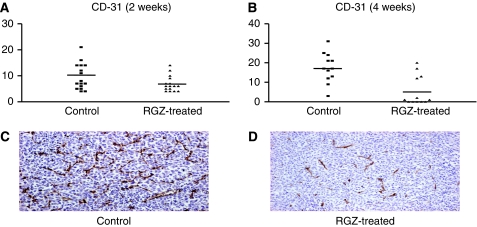
Immunohistochemistry (IHC) for CD-31 evaluation in tumour tissues from control (i.e., solvent treated) and rosiglitazone (RGZ)-treated (**A**, 2 weeks; **B**, 4 weeks) mice. *Y* axis: number of CD-31-positive cells per field. (**C**) Example of a field showing high CD-31 staining in the tissue sample from a control mouse. (**D**) Example of a field showing a few CD-31-positive cells after 4 weeks of treatment with RGZ ( × 100 magnification).

In the same tumour samples used for apoptosis assessment, tumour angiogenesis was investigated by determining the percentage of cells showing a positive staining for CD-31. The number of immunopositive cells seemed reduced (yet not significantly, *P*=0.058) after 2 weeks of RGZ treatment (Table 1 and Figure 6A). In tumours of mice treated with RGZ for 4 weeks, the reduction in CD-31-positive cells became statistically significant (*P*<0.005) (Table 1 and Figure 6B). A large variability among the different tumour samples of RGZ-treated mice was observed, and a strong inhibitory effect could be observed in some of them (Figures 6C–D).

### PPAR*γ* expression

**Figure 7:**
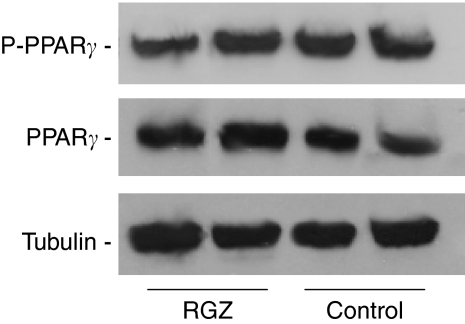
Western blot analysis of the amount of peroxisome proliferator-activated receptor *γ* (PPAR*γ*) and phosphorylated PPAR*γ* (P-PPAR*γ*) in rosiglitazone (RGZ)-treated and solvent-treated mice. Tubulin amount was determined to verify equivalent protein loading.

To determine whether RGZ treatment affected the expression of PPAR*γ*, both mRNA and protein levels were evaluated. The PPAR*γ* transcript, as assessed by quantitative real-time reverse transcriptase PCR, did not significantly differ in tumours from control or RGZ-treated (4 weeks) mice (Table 2). Similarly, the amount of PPAR*γ* protein, detected by western blot analysis, was not different in the two groups. Because there is evidence that PPAR*γ* phosphorylation reduces the activity of the receptor (Shao *et al*, 1998), western blot analysis was performed using an anti-phosphoserinel antibody as well. Moreover, the amount of phosphorylated, hence inactivated, PPAR*γ* was virtually equal in tumours from RGZ-treated and control mice (Table 2). A representative example of PPAR*γ* and phosphorylated PPAR*γ* expression in RGZ-treated and solvent-treated mice is shown in Figure 7.

### MMP-9 and TIMP-1 expression

We demonstrated previously that RGZ determines a significant reduction in MMP-9 and a trend towards an increase in TIMP-1 expression in SK-N-AS cells (Cellai *et al*, 2006). In this study, we were able to determine the amount of mRNA of MMP-9 and TIMP-1 in the few residual tumour tissues that were available (*n*=3 for each group, i.e., RGZ-treated (4 weeks) or control mice). Owing to the limited number of samples and the variability of data in each group, a statistical analysis could not be performed. However, the median of the amount of MMP-9 mRNA seemed to be reduced in RGZ-treated mice compared with control animals (0.83 *vs* 0.94 (2^−ΔΔCt^) × 10^3^, respectively), whereas the expression of TIMP-1 showed an opposite trend (3.78 *vs* 2.13 (2^−ΔΔCt^) × 10^3^, respectively).

## Discussion

We demonstrated previously that RGZ exerts anti-neoplastic effects in SK-N-AS NB cells in which a transcriptionally active PPAR*γ* is present. In this new study, we evaluated the effect of RGZ against SK-N-AS cells xenografted *in vivo*. Rosiglitazone was administered for 4 weeks at a dose of 150 mg kg^−1^ day^−1^, in agreement with previously published data, reporting doses ranging from 120 to 150 mg kg^−1^ daily (Heaney *et al*, 2002; Bogazzi *et al*, 2004). According to the low toxicity profile shown by RGZ as an anti-diabetic agent (Yki-Järvinen, 2004), RGZ was well tolerated in our study and no body weight loss or lethal toxicity was observed during the treatment. At the end of the scheduled time for RGZ administration, no difference in plasma glucose levels was detected, suggesting that RGZ may be administered in non-diabetic subjects without undesirable effects on glucose metabolism. With regard to tumour growth, a significant difference between the TW of RGZ-treated and control mice was observed (TWI=70%). It is noteworthy that three out of eight tumours grown in mice exposed to RGZ weighed <50 mg at the end of treatment. These animals were monitored for an additional period of time. In two cases, tumour re-growth was observed only 2 weeks after the end of treatment, and in one case, the tumour never started to re-grow. Therefore, in about 40% of mice, RGZ administration determined a virtually complete inhibitory effect on NB growth that lasted, at least partially, even after the cessation of treatment. Thus, this PPAR*γ* agonist proved to effectively inhibit the growth of an NB tumour xenograft, in keeping with similar findings obtained using PPAR*γ* agonists against other types of tumour xenografts, such as colorectal (Katoh *et al*, 2004; Yoshizumi *et al*, 2004; Cesario *et al*, 2006; Shimazaki *et al*, 2008), lung (Nemenoff, 2007; Hazra *et al*, 2008; Roman, 2008), prostate (Annicotte *et al*, 2006), bladder and ovarian cancer (Kassouf *et al*, 2006; Xin *et al*, 2007).

To elucidate the mechanism(s) by which RGZ exerted an inhibitory effect on tumour growth, tissue samples were obtained from killed RGZ-treated and control mice after 2 and 4 weeks of treatment. After 2 weeks, in tumours excised from animals treated with RGZ, the number of apoptotic cells seemed to be increased, although the difference was not statistically significant. This result is in agreement with our previous *in vitro* observations on SK-N-AS cells exposed to RGZ (Cellai *et al*, 2006), as well as with other similar reports on the pro-apoptotic effects of PPAR*γ* agonists in different NB cell lines (Rohn *et al*, 2001; Kato *et al*, 2002; Kim *et al*, 2003; Schultze *et al*, 2006). The PPAR*γ* agonists-induced apoptosis has been described as a surveillance mechanism against tumour growth and invasion in NB (Grommes *et al*, 2004; Stupack *et al*, 2006). Conversely, at the end of treatment, the number of apoptotic cells was significantly higher in tumours from control mice, most likely because of the presence of large expanding masses of about 2 g in these tumours. Accordingly, we observed a direct relationship between TW and apoptotic cells in tumours grown in solvent-treated mice. The finding of a high percentage of apoptotic cells in growing lesions is a known phenomenon that has been described in tumours of different tissues, such as the breast (Vakkala *et al*, 1999; Wong *et al*, 2001; O’Donovan *et al*, 2003) and the adrenal gland (Bernini *et al*, 2002; Stojadinovic *et al*, 2003; Luciani *et al*, 2004). Anyway, when we compared the rate of apoptosis in tumour samples of the same size from solvent-treated or RGZ-treated mice, we observed a markedly higher percentage of apoptotic cells in the latter group, thus confirming that a pro-apoptotic effect of RGZ on NB cells occurs *in vivo* as well. We also observed a moderately reduced percentage of Ki-67-positive NB cells in RGZ-treated mice *vs* control mice, both after 2 and 4 weeks of treatment. Also, in this case, the presence of large volume tumours with extended necrotic areas in control animals at the end of the treatment period may likely justify the absence of a greater difference in the proliferation rate between the two groups of animals.

Similar to many other tumours, the progression of NB is associated with the activity of MMPs that promote the invasion of the extra-cellular matrix by tumoural cells and trigger angiogenesis by different modalities, such as the release of vascular endothelial growth factor, the recruitment of pericytes along endothelial cells and the mobilisation of haematopoietic stem cells into circulation (Sugiura *et al*, 1998; Bergers *et al*, 2000; Heissig *et al*, 2002; Chantrain *et al*, 2004). We previously demonstrated the efficacy of RGZ in inhibiting *in vitro* SK-N-AS cell invasiveness through a significant reduction in the expression of MMP-9 (Cellai *et al*, 2006). In this study, we have shown that RGZ determines a marked anti-angiogenic effect *in vivo*, as assessed by the significant reduction in the number of CD-31 immunopositive cells. In addition, in residual tumour samples, the amount of MMP-9 mRNA seemed to be reduced at the end of RGZ treatment compared with that in the control group, whereas an opposite trend was observed for TIMP-1 expression. It has to be said that data on MMP-9 and TIMP-1 expression were obtained from a limited sample size and it was not possible to perform a statistical analysis. Nevertheless, these observations seem to be in agreement with the observed anti-angiogenic effect of RGZ *in vivo* and may be responsible, at least partially, for such an effect.

Finally, we demonstrated that neither the expression of PPAR*γ* nor the expression of the inactivated form of the receptor, that is, phosphorylated PPAR*γ*, were significantly different in tumours from RGZ-treated and solvent-treated mice, thus suggesting that the binding capacity of this molecule to its receptor was maintained throughout the entire treatment period. However, we cannot exclude the fact that the anti-neoplastic effects of RGZ were, at least in part, PPAR*γ* independent. Previous *in vitro* and *in vivo* evidence suggests that the anti-proliferative effect of TZD is independent of PPAR*γ* transcriptional activity (Galli *et al*, 2006). Palakurthi *et al* (2001) have shown that TZD induces growth arrest by inhibition of translation initiation in PPAR*γ*^−/−^ embryonic stem cells. Furthermore, TZD analogues, which have a double bond adjoining the terminal TZD ring that is responsible for the abrogation of the PPAR*γ* ligand property, retain the ability to induce apoptosis in prostate cancer cells (Weng *et al*, 2006).

In conclusion, in this study, we demonstrated that the PPAR*γ* agonist RGZ effectively inhibits tumour growth in a human NB xenograft. This anti-tumoural effect seemed to be mostly due to a strong anti-angiogenic activity. To our knowledge, this is the first demonstration that PPAR*γ* agonists may be effective against NB *in vivo*, thus suggesting the interest for clinical studies. It has to be said that SK-N-AS cells were selected for this study on the basis of the presence of a high transactivation potential of PPAR*γ*, which was related to low levels of phosphorylated PPAR*γ* (Luciani *et al*, 2004). Thus, in designing clinical trials, it might be useful to preliminarily determine the ratio between total and phosphorylated PPAR*γ* in tissue specimens to select hypothetically responsive patients.

## Figures and Tables

**Table 1:** Anti-tumour effects of oral rosiglitazone maleate delivered daily against the SK-N-AS human neuroblastoma xenograft.

			**Number of apoptotic cells**^b^ **(mean value±s.e.)**		**MVD**^c^ **(mean value±s.e.)**	
**Drug**	**Dose (mg kg^−1^)**	**TWI%** a	**2 w** d	**4 w** d	**2 w** d	**4 w** d
Solvent^e^	—	—	9±2	48±7	10±1	17±2
Rosiglitazone	150	70°	14±2	28±7^*^	7±1	5±2^**^

a TWI%=tumour weight inhibition percentage in rosiglitazone-treated *vs* control mice. TWI% was calculated 1 day after the last treatment (4 weeks after tumour inoculum).

b Mean number of apoptotic (caspase-3+) cells per field ±s.e. ( × 400 magnification).

c MVD=microvessel density. MVD=mean number of CD31^+^ cells per field ±s.e. ( × 400 magnification).

d 2 w and 4 w=2 weeks and 4 weeks after the beginning of treatment.

e Solvent=glycine/HCl buffer at pH 2.3.

°*P*<0.005 *vs* controls, by analysis of variance followed by Bonferroni test. ^*^P<0.05 and ^**^P<0.005 *vs* controls, by Student's *t*-test (two-tailed).

**Table 2:** PPAR*γ* expression in RGZ-treated or solvent-treated mice

	**Solvent**	**RGZ**
PPAR*γ* (mRNA expression)^a^	4.91±1.12	2.295±0.815
PPAR*γ* (total protein amount)^b^	193.15±1.53	192.82±4.44
P-PPAR*γ* (protein amount)^b^	167.495±20.60	175.125±18.92

Abbreviations: PPAR*γ=*peroxisome proliferator-activated receptor *γ*; P-PPAR*γ=*phosphorylated PPAR*γ*; RGZ=rosiglitazone.

a (2^−ΔΔCt^) × 10^3^.

b Arbitrary units (as assessed by densitometry).

## References

[bib1] Annicotte JS, Iankova I, Miard S, Fritz V, Sarruf D, Abella A, Berthe ML, Noël D, Pillon A, Iborra F, Dubus P, Maudelonde T, Culine S, Fajas L (2006) Peroxisome proliferator-activated receptor gamma regulates E-cadherin expression and inhibits growth and invasion of prostate cancer. Mol Cell Biol 26: 7561–7574 17015477 10.1128/MCB.00605-06 PMC1636859

[bib2] Bergers G, Brekken R, McMahon G, Vu TH, Itoh T, Tamaki K, Tanzawa K, Thorpe P, Itohara S, Werb Z, Hanahan D (2000) Matrix metalloproteinase-9 triggers the angiogenic switch during carcinogenesis. Nat Cell Biol 2: 737–744 11025665 10.1038/35036374 PMC2852586

[bib3] Bernini GP, Moretti A, Viacava P, Bonadio AG, Iacconi P, Miccoli P, Salvetti A (2002) Apoptosis control and proliferation marker in human normal and neoplastic adrenocortical tissues. Br J Cancer 86: 1561–1565 12085205 10.1038/sj.bjc.6600287 PMC2746588

[bib4] Bogazzi F, Ultimieri F, Raggi F, Russo D, Vanacore R, Guida C, Viacava P, Cecchetti D, Acerbi G, Brogioni S, Cosci C, Gasperi M, Bartalena L, Martino E (2004) PPARgamma inhibits GH synthesis and secretion and increases apoptosis of pituitary GH-secreting adenomas. Eur J Endocrinol 150: 863–875 15191358 10.1530/eje.0.1500863

[bib5] Burstein HG, Demetri GD, Mueller E, Sarraf P, Spiegelman BM, Winer EP (2003) Use of peroxisome proliferator-activated receptor (PPAR) gamma ligand troglitazone as treatement for refractory breast cancer: a phase II study. Breast Cancer Res Treat 79: 391–397 12846423 10.1023/a:1024038127156

[bib6] Cellai I, Benvenuti S, Luciani P, Galli A, Ceni E, Simi L, Baglioni S, Muratori M, Ottanelli B, Serio M, Thiele CJ, Peri A (2006) Antineoplastic effects of rosiglitazone and PPARgamma transactivation in neuroblastoma cells. Br J Cancer 95: 879–888 16969347 10.1038/sj.bjc.6603344 PMC2360542

[bib7] Cesario RM, Stone J, Yen WC, Bissonnette RP, Lamph WW (2006) Differentiation and growth inhibition mediated via the RXR: PPARgamma heterodimer in colon cancer. Cancer Lett 240: 225–233 16271436 10.1016/j.canlet.2005.09.010

[bib8] Chantrain CF, Shimada H, Jodele S, Groshen S, Ye W, Shalinsky DR, Werb Z, Coussens LM, DeClerck YA (2004) Stromal matrix metalloproteinase-9 regulates the vascular architecture in neuroblastoma by promoting pericyte recruitment. Cancer Res 64: 1675–1686 14996727 10.1158/0008-5472.can-03-0160

[bib9] Chattopadhyay N, Singh DP, Heese O, Godbole MM, Sinohara T, Black PM, Brown EM (2000) Expression of peroxisome proliferator-activated receptors (PPARS) in human astrocytic cells: PPARgamma agonists as inducers of apoptosis. J Neurosci Res 61: 67–74 10861801 10.1002/1097-4547(20000701)61:1<67::AID-JNR8>3.0.CO;2-7

[bib10] Cioppi F, Simi L, Luciani P, Petraglia F, Susini T, Cobellis L, Serio M, Maggi M, Peri A (2004) Expression of uteroglobin and matrix metalloproteinase-9 genes in endometrial cancer: relationship to estrogen and progesterone receptor status. Oncol Rep 11: 427–433 14719079

[bib11] De Bernardi B, Nicolas B, Boni L, Indolfi P, Carli M, Cordero Di Montezemolo L, Donfrancesco A, Pession A, Provenzi M, di Cataldo A, Rizzo A, Tonini GP, Dallorso S, Conte M, Gambini C, Garaventa A, Bonetti F, Zanazzo A, D'Angelo P, Bruzzi P (2003) Disseminated neuroblastoma in children older than one year at diagnosis: comparable results with three consecutive high-dose protocols adopted by the Italian Co-Operative Group for Neuroblastoma. J Clin Oncol 21: 1592–1601 12697885 10.1200/JCO.2003.05.191

[bib12] Debrock G, Vanhentenrijk V, Sciot R, Debiec-Rychter M, Oyen R, Van Oosterom A (2003) A phase II trial with rosiglitazone in liposarcoma patients. Br J Cancer 89: 1409–1412 14562008 10.1038/sj.bjc.6601306 PMC2394353

[bib13] Demetri GD, Fletcher CD, Mueller E, Sarraf P, Naujoks R, Campbell N, Spiegelman BM, Singer S (1999) Induction of solid tumor differentiation by the peroxisome proliferator-activated receptor-*γ* ligand troglitazone in patients with liposarcoma. Proc Natl Acad Sci USA 96: 3951–3956 10097144 10.1073/pnas.96.7.3951 PMC22401

[bib14] Desvergne B, Whali W (1999) Peroxisome proliferator-activated receptors: nuclear control of metabolism. Endocrine Rev 20: 649–688 10529898 10.1210/edrv.20.5.0380

[bib15] Emmans VC, Rodway HA, Hunt AN, Lillycrop KA (2004) Regulation of cellular processes by PPARgamma ligands in neuroblastoma cells is modulated by the level of retinoblastoma protein expression. Biochem Soc Trans 32: 840–842 15494029 10.1042/BST0320840

[bib16] Galli A, Mello T, Ceni E, Surrenti E, Surrenti C (2006) The potential of antidiabetic thiazolidinediones for anticancer therapy. Expert Opin Investig Drugs 15: 1039–1049 10.1517/13543784.15.9.1039 16916271

[bib17] Garaventa A, Luksch R, Lo Piccolo MS, Cavadini E, Montaldo PG, Pizzitola MR, Boni L, Ponzoni M, Decensi A, De Bernardi B, Bellani FF, Formelli F (2003) Phase I trial and pharmacokinetics of fenretinide in children with neuroblastoma. Clin Cancer Res 9: 2032–2039 12796365

[bib18] Grommes C, Landreth GE, Heneka MT (2004) Antineoplastic effects of peroxisome proliferator-activated receptor gamma agonists. Lancet Oncol 5: 419–429 15231248 10.1016/S1470-2045(04)01509-8

[bib19] Han SW, Greene ME, Pitts J, Wada RK, Sidell N (2001) Novel expression and function of peroxisome proliferator-activated receptor gamma (PPARgamma) in human neuroblastoma cells. Clin Cancer Res 7: 98–104 11205925

[bib20] Hazra S, Peebles KA, Sharma S, Mao JT, Dubinett SM (2008) The role of PPARgamma in the cyclooxygenase pathway in lung cancer. PPAR Res 2008: 790568 18769553 10.1155/2008/790568 PMC2526169

[bib21] Heaney AP, Fernando M, Yong WH, Melmed S (2002) Functional PPAR-gamma receptor is a novel therapeutic target for ACTH-secreting pituitary adenomas. Nat Med 8: 1281–1287 12379847 10.1038/nm784

[bib22] Heissig B, Hattori K, Dias S, Friedrich M, Ferris B, Hackett NR, Crystal RG, Besmer P, Lyden D, Moore MA, Werb Z, Rafii S (2002) Recruitment of stem and progenitor cells from the bone marrow niche requires MMP-9 mediated release of kit-ligand. Cell 109: 625–637 12062105 10.1016/s0092-8674(02)00754-7 PMC2826110

[bib23] Hisatake J, Ikezoe T, Carey Y, Holden S, Tomoyasu S, Koeffler HP (2000) Down regulation of prostate-specific antigen expression by ligands for peroxisome-activator receptor gamma in human prostate cancer. Cancer Res 60: 5494–5498 11034093

[bib24] Hsu SM, Raine L, Fanger H (1981) Use of avidin-biotin peroxidase complex (ABC) in immunoperoxidase techniques: a comparation between ABC and unlabeled antibody (PAP) procedures. J Histochem Cytochem 29: 577–580 6166661 10.1177/29.4.6166661

[bib25] Jiang C, Ting AT, Seed B (1998) PPAR-gamma agonists inhibit production of monocyte inflammatory cytokines. Nature 391: 82–86 9422509 10.1038/34184

[bib26] Joshi VV, Tsongalis GJ (1997) Correlation between morphologic and nonmorphologic prognostic markers of neuroblastoma. Ann N Y Acad Sci 824: 71–83 9382456 10.1111/j.1749-6632.1997.tb46210.x

[bib27] Jude-Aubry C, Pernin A, Favez T, Burger AG, Wahli W, Meier CA, Desvergne B (1997) DNA binding properties of peroxisome proliferator activated receptor subtypes on various natural peroxisome proliferator response elements. J Biol Chem 272: 25252–25259 9312141 10.1074/jbc.272.40.25252

[bib28] Kassouf W, Chintharlapalli S, Abdelrahim M, Nelkin G, Safe S, Kamat AM (2006) Inhibition of bladder tumor growth by 1,1-bis(3′-indolyl)-1-(p-substitutedphenyl) methanes: a new class of peroxisome proliferator-activated receptor gamma agonists. Cancer Res 66: 412–418 16397256 10.1158/0008-5472.CAN-05-2755

[bib29] Kato M, Nagaya T, Fujieda M, Saito K, Yoshida J, Seo H (2002) Expression of PPAR*γ* and its ligand-dependent growth inhibition in human brain tumor cell lines. Jpn J Cancer Res 93: 660–666 12079514 10.1111/j.1349-7006.2002.tb01304.x PMC5927044

[bib30] Katoh M, Feldhaus S, Schnitzer T, Bauer S, Schumacher U (2004) Limited tumor growth (HT29) *in vivo* under RO205-2349 is due to increased apoptosis and reduced cell volume but not to decreased proliferation rate. Cancer Lett 210: 7–15 15172115 10.1016/j.canlet.2004.01.010

[bib31] Kersten S, Desvergne B, Wahli W (2000) Roles of PPARs in health and disease. Nature 405: 421–424 10839530 10.1038/35013000

[bib32] Kim EJ, Park KS, Chung SY, Sheen YY, Moon DC, Song YS, Kim KS, Song S, Yun YP, Lee MK, Oh KW, Yoon DY, Hong JT (2003) Peroxisome proliferator-activated receptor-*γ* activator 15-deoxy-Δ12,14- prostaglandin J2 inhibits neuroblastoma cell growth through induction of apoptosis: association with extracellular signal regulated kinase signal pathway. J Pharmacol Exp Ther 307: 505–517 12966153 10.1124/jpet.103.053876

[bib33] Koeffler HP (2003) Peroxisome proliferator-activated receptor *γ* and cancers. Clin Cancer Res 9: 1–9 12538445

[bib34] Kulke MH, Demetri GD, Sharpless NE, Ryan DP, Shivdasani R, Clark JS, Spiegelman BM, Kim H, Mayer RJ, Fuchs CS (2002) A phase II study of troglitazone, an activator of PPAR gamma receptor, in patients with chemoteraphy-resistant matastatic colorectal cancer. Cancer J 8: 395–399 12416897 10.1097/00130404-200209000-00010

[bib35] Luciani P, Ferruzzi P, Arnaldi G, Crescioli C, Benvenuti S, Nesi G, Valeri A, Greeve I, Serio M, Mannelli M, Peri A (2004) Expression of the novel adrenocorticotropin-responsive gene selective Alzheimer's disease indicator-1 in the normal adrenal cortex and in adrenocortical adenomas and carcinomas. J Clin Endocrinol Metab 89: 1332–1339 15001630 10.1210/jc.2003-031065

[bib36] Maris JM, Hogarty MD, Bagatell R, Cohn SL (2007) Neuroblastoma. Lancet 369: 2106–2120 17586306 10.1016/S0140-6736(07)60983-0

[bib37] Matthay KK, Villablanca JG, Seeger RC, Stram DO, Harris RE, Ramsay NK, Swift P, Shimada H, Black CT, Brodeur GM, Gerbing RB, Reynolds CP (1999) Treatment of high-risk neuroblastoma with intensive chemotherapy, radiotherapy, autologous bone marrow transplantation, and 13-cis-retinoic acid. Children's Cancer Group. N Engl J Med 341: 1165–1173 10519894 10.1056/NEJM199910143411601

[bib38] Melino G, Thiele CJ, Knight RA, Piacentini M (1997) Retinoids and the control of growth/death decisions in human neuroblastoma cell lines. J Neurooncol 31: 65–83 9049832 10.1023/a:1005733430435

[bib39] Morosetti R, Servidei T, Mirabella M, Rutella S, Mangiola A, Maira G, Mastrangelo R, Koeffler HP (2004) The PPARgamma ligands PGJ2 and rosiglitazone show a differential ability to inhibit proliferation and to induce apoptosis and differentiation of human glioblastoma cell lines. Int J Oncol 25: 493–502 15254749

[bib40] Mueller E, Smith M, Sarraf P, Kroll T, Aiyer A, Kaufman DS, Oh W, Demetri G, Figg WD, Zhou XP, Eng C, Spiegelman BM, Kantoff PW (2000) Effects of ligand activation of peroxisome proliferators-activated receptor *γ* in human prostate cancer. Procl Natl Acad Sci USA 97: 10990–10995 10.1073/pnas.180329197 PMC27136 10984506

[bib41] National Cancer Institute, Surveillance, Epidemiology and End Results Database. Available at http://seer.cancer.gov Accessed on November, 2005

[bib42] Nemenoff RA (2007) Peroxisome proliferator-activated receptor-gamma in lung cancer: defining specific versus ‘off-target’ effectors. J Thorac Oncol 2: 989–992 17975488 10.1097/JTO.0b013e318158cf0a

[bib43] Nwankwo JO, Robbins ME (2001) Peroxisome proliferator-activated receptor gamma expression in human malignant and normal brain, breast and prostate-derived cells. Prostaglandins Leukot Essent Fatty Acids 64: 241–245 11418018 10.1054/plef.2001.0266

[bib44] O’Donovan N, Crown J, Stunnel H, Hill AD, McDermott E, O’Higgins N, Duffy MJ (2003) Caspase 3 in breast cancer. Clin Cancer Res 9: 738–742 12576443

[bib45] Palakurthi SS, Aktas H, Grubissich LM, Mortensen RM, Halperin JA (2001) Anticancer effects of thiazolidinediones are independent of peroxisome proliferator-activated receptor *γ*and mediated by inhibition of translation initiation. Cancer Res 61: 6213–6218 11507074

[bib46] Peri A, Cellai I, Benvenuti S, Luciani P, Baglioni S, Serio M (2008) PPARgamma in neuroblastoma. PPAR Res 2008: 917815 18528516 10.1155/2008/917815 PMC2397449

[bib47] Pratesi G, Pezzoni G, Parmiani G (1985) NMU-1, a new transplantable mouse lung tumor: biological and chemosensitivity properties *in vivo*. Cancer Treat Rep 69: 219–221 3971393

[bib48] Reginato MJ, Bailey ST, Krakow SL, Minami C, Ishii S, Tanaka H, Lazar MA (1998) A potent antidiabetic thiazolidinedione with unique peroxisome proliferator-activated receptor *γ*-activating properties. J Biol Chem 273: 32679–32684 9830009 10.1074/jbc.273.49.32679

[bib49] Reynolds CP, Matthay KK, Villablanca JG, Maurer BJ (2003) Retinoid therapy of high-risk neuroblastoma. Cancer Lett 197: 185–192 12880980 10.1016/s0304-3835(03)00108-3

[bib50] Ricote M, Huang J, Fajas L, Li A, Welch J, Najib J, Witztum JL, Auwerx J, Palinski W, Glass CK (1998) Expression of the peroxisome proliferator-activated receptor gamma (PPAR gamma) in human atherosclerosis and regulation in macrophages by colony stimulating factors and oxidized low density protein. Procl Natl Acad Sci USA 95: 7614–7619 10.1073/pnas.95.13.7614 PMC22700 9636198

[bib51] Rohn TT, Wong SM, Cotman CW, Cribbs DH (2001) 15-deoxy-Δ12,14-prostaglandin J2, a specific ligand for peroxisome proliferator-activated receptor-*γ*, induces neuronal apoptosis. Neuroreport 12: 839–843 11277593 10.1097/00001756-200103260-00043

[bib52] Roman J (2008) Peroxisome proliferator-activated receptor gamma and lung cancer biology: implications for therapy. J Investig Med 56: 528–533 10.2310/JIM.0b013e3181659932 18317436

[bib53] Saltiel AR, Olefsky JM (1996) Thiazolidinediones in the treatment of insulin resistance and type II diabetes. Diabetes 45: 1661–1669 8922349 10.2337/diab.45.12.1661

[bib54] Schultze K, Böck B, Eckert A, Oevermann L, Ramacher D, Wiestler O, Roth W (2006) Troglitazone sensitizes tumor cells to TRAIL-induced apoptosis via down-regulation of FLIP and Survivin. Apoptosis 11: 1503–1512 16820965 10.1007/s10495-006-8896-3

[bib55] Servidei T, Morosetti R, Ferlini C, Cusano G, Scambia G, Mastrangelo R, Koeffler HP (2004) The cellular response to PPARgamma ligands is related to phenotype of neuroblastomi cell lines. Oncol Res 14: 345–354 15301425 10.3727/0965040041292297

[bib56] Shao D, Rangwala SM, Bailey ST, Krakow SL, Reginato MJ, Lazar MA (1998) Interdomain communication regulating ligand binding by PPAR-gamma. Nature 396: 377–380 9845075 10.1038/24634

[bib57] Shimazaki N, Togashi N, Hanai M, Isoyama T, Wada K, Fujita T, Fujiwara K, Kurakata S (2008) Anti-tumor activity of CS-7017, a selective peroxisome proliferator-activated receptor gamma agonist of thiazolidinedione class, in human tumor xenografts and a syngeneic tumour implant model. Eur J Cancer 44: 1734–1743 18511262 10.1016/j.ejca.2008.04.016

[bib58] Spiegelman BM (1998) PPAR-gamma: adipogenic regulator and thiazolidinedione receptor. Diabetes 47: 507–514 9568680 10.2337/diabetes.47.4.507

[bib59] Stojadinovic A, Brennan MF, Hoos A, Omeroglu A, Leung DH, Dudas ME, Nissan A, Cordon-Cardo C, Ghossein RA (2003) Adrenocortical adenoma and carcinoma: histopathological and molecular comparative analysis. Mod Pathol 16: 742–751 12920217 10.1097/01.MP.0000081730.72305.81

[bib60] Stupack DG, Teitz T, Potter MD, Mikolon D, Houghton PJ, Kidd VJ, Lahti JM, Cheresh DA (2006) Potentiation of neuroblastoma metastasis by loss of caspase-8. Nature 439: 95–99 16397500 10.1038/nature04323

[bib61] Sugiura Y, Shimada H, Seeger RC, Laug WE, DeClerck YA (1998) Matrix metalloproteinases-2 and -9 are expressed in human neuroblastoma: contribution of stromal cells to their production and correlation with metastasis. Cancer Res 58: 2209–2216 9605768

[bib62] Tontonoz P, Kim JB, Graves RA, Spiegelman BM (1993) ADD1: a novel helix-loop-helix transcription factor associated with adipocytes determination and differentiation. Mol Cell Biol 13: 4753–4759 8336713 10.1128/mcb.13.8.4753-4759.1993 PMC360101

[bib63] Tsubouchi Y, Sano H, Kawahito Y, Mukai S, Yamada R, Kohno M, Inoue K, Hla T, Kondo M (2000) Inhibition of human lung cancer-cell growth by peroxisome proliferators-activated receptor gamma agonist through induction of apoptosis. Biochem Biophys Res Commun 270: 400–405 10753637 10.1006/bbrc.2000.2436

[bib64] Vakkala M, Pääkö P, Soini Y (1999) Expression of caspases 3, 6, and 8 is increased in parallel with apoptosis and histological aggressiveness of the breast lesion. Br J Cancer 81: 592–599 10574243 10.1038/sj.bjc.6690735 PMC2362889

[bib65] Valentiner U, Carlsson L, Erttmann RM, Hildebrandt H, Shumacher U (2005) Ligands for peroxisome proliferator-activated receptor-*γ* have inhibitory effects on growth of human neuroblastoma cells *in vitro*. Toxicology 213: 157–168 16009482 10.1016/j.tox.2005.05.024

[bib66] Vecchi A, Garlanda C, Lampugnani MG, Resnati M, Matteucci C, Stoppacciaro A, Schnurch H, Risau W, Ruco L, Mantovani A (1994) Monoclonal antibodies specific for endothelial cells of mouse blood vessels. Their application in the identification of adult and embryonic endothelium. Eur J CellBiol 63: 247–254 8082649

[bib67] Voigt A, Zintl F (2003) Effects of retinoic acid on proliferation, apoptosis, citotoxicity migration, and invasion of neuroblastoma cells. Med Pediatr Oncol 40: 205–213 12555246 10.1002/mpo.10250

[bib68] Weng JR, Chen CY, Pinzone JJ, Ringel MD, Chen CS (2006) Beyond peroxisome proliferator-activated receptor gamma signaling: the multi-facets of the antitumor effect of thiazolidinediones. Endocr Relat Cancer 13: 401–413 16728570 10.1677/erc.1.01182

[bib69] Wong SC, Chan JK, Lee KC, Hsiao WL (2001) Differential expression of p16/p21/p27 and cyclin D1/D3, and their relationships to cell proliferation, apoptosis, and tumor progression in invasive ductal carcinoma of the breast. J Pathol 194: 35–42 11329139 10.1002/path.838

[bib70] Workman P, Balmain A, Hickman JA, McNally NJ, Rohas AM, Mitchison NA, Pierrepoint CG, Raymond R, Rowlatt C, Stephens TC (1988) UKCCCR guidelines for the welfare of animals in experimental neoplasia. Lab Anim 22: 195–201 3172698 10.1258/002367788780746467

[bib71] Xin B, Yokoyama Y, Shigeto T, Futagami M, Mizunuma H (2007) Inhibitory effect of meloxicam, a selective cyclooxygenase-2 inhibitor, and ciglitazone, a peroxisome proliferator-activated receptor gamma ligand, on the growth of human ovarian cancers. Cancer 110: 791–800 17582802 10.1002/cncr.22854

[bib72] Yki-Järvinen H (2004) Thiazolidinediones. N Engl J Med 351: 1016–1018 10.1056/NEJMra041001 15356308

[bib73] Yoshizumi T, Ohta T, Ninomiya I, Terada I, Fushida S, Fujimura T, Nishimura G, Shimizu K, Yi S, Miwa K (2004) Thiazolidinedione, a peroxisome proliferator-activated receptor-gamma ligand, inhibits growth and metastasis of HT-29 human colon cancer cells through differentiation-promoting effects. Int J Oncol 25: 631–639 15289864

